# Electrochemical Sunset Yellow Biosensor Based on Photocured Polyacrylamide Membrane for Food Dye Monitoring

**DOI:** 10.3390/s18010101

**Published:** 2018-01-01

**Authors:** Normazida Rozi, Amalina Ahmad, Lee Yook Heng, Loh Kee Shyuan, Sharina Abu Hanifah

**Affiliations:** 1 School of Chemical Sciences and Food Technology, Faculty of Science and Technology, Universiti Kebangsaan Malaysia, Bangi 43600, Selangor, Malaysia; adizam_92@yahoo.com (N.R.); maynotemus@yahoo.com (A.A.); leeyookheng@yahoo.co.uk (L.Y.H.); 2 Fuel Cell Institute, Universiti Kebangsaan Malaysia, Bangi 43600, Selangor, Malaysia; ksloh@ukm.edu.my; 3 Polymer Research Center, Faculty of Science and Technology, Universiti Kebangsaan Malaysia, Bangi 43600, Selangor, Malaysia

**Keywords:** electrochemical biosensor, Sunset Yellow, synthetic food dye, laccase, photopolymerization

## Abstract

An enzyme-based electrochemical biosensor was investigated for the analysis of Sunset Yellow synthetic food dye. A glassy carbon electrode was coated with a poly(acrylamide-*co*-ethyl methacrylate) membrane to immobilize laccase using a single-step photopolymerization procedure. Poly(acrylamide-*co*-ethyl methacrylate) membrane was demonstrated to have acceptable water absorption and suitable for biosensor application. Sunset Yellow biosensor exhibited a linear response range from 0.08 to 10.00 µM with a detection limit of 0.02 µM. This biosensor was successfully used to determine Sunset Yellow in soft drinks with recoveries of 99.0–101.6%. The method was validated using high-performance liquid chromatography, indicating the biosensor can be as a promising alternative method for Sunset Yellow detection.

## 1. Introduction

In recent years, the use of synthetic dyes in food and drink has become an important food safety issue because of their effects on human health. Synthetic dyes have been used to improve and maintain the appearance, color, and texture of food during processing and storage [1,2]. However, if the intake of a dye exceeds a certain threshold then it may be pathogenic [3]. Sunset Yellow contains an azo functional group (N=N) and an aromatic ring structure, which can be harmful to human health and cause hyperactivity in children [4,5,6,7]. Excessive intake can also result in allergies, asthma, and migraines [8]. Moreover, studies have shown that azo dyes can cause bladder cancer in men and hepatocarcinoma in mice [9]. Nevertheless, Sunset Yellow is frequently used in food products because it is less expensive and more stable than natural dyes. Therefore, synthetic food dyes are restricted by the United Nations’ Food and Agricultural Organization (FAO) and World Health Organization (WHO) [10]. According to the WHO, the accepted daily intake (ADI) value for Sunset Yellow is 0–4 mg kg^−1^ [11]. Moreover, the concentration of Sunset Yellow in non-alcoholic beverages should not exceed 50 mg L^−1^ [1].

In order to regulate food quality and provide safety assurances for consumers, appropriate and effective methods to detect synthetic dyes are indispensable [5,12]. Various methods have been used for food dyes analyses, including spectroscopy [1], high-performance liquid chromatography (HPLC) [13], liquid-liquid extraction (LLE) [14] and electrophoresis [15]. However, these complicated methods do not permit the rapid detection of food dyes [4,16]. Several chemosensor devices have been reported for the analysis of food dyes. Yu et al. [17] reported a glassy carbon electrode (GCE) modified with platinum nanoparticles and a cetrimonium bromide (CTAB)/graphene composite for Sunset Yellow analysis. Ghoreishi et al. [2] used a screen-printed electrode modified with gold nanoparticles for the same purpose. Recently, Wang et al. [4] reported a GCE based on polypyrrole/single-carbon nanotubes for Sunset Yellow determination. Although these chemosensors enabled the rapid analysis of Sunset Yellow, they exhibited higher redox potential. At higher potentials, the sensor was vulnerable to interference from many other electroactive substances.

In the present study, a Sunset Yellow biosensor was developed by immobilizing laccase in a photocurable poly(acrylamide-*co*-ethylmethacrylate) (AAm-*co*-EMA) membrane. AAm is a biocompatible, water-soluble polymer which can be used directly as an enzyme support [18,19,20]. EMA is a hydrophobic polymer that is used as a peelable membrane [21,22,23] and to minimize enzyme leaching [24] by controlling the hydrophilicity of membrane. Previously, laccase was used as a biosensor for the detection of commercially reactive dyes [18]; methyl orange, an azo dye, was used as a model and compared with other commercially reactive dyes. To the best of our knowledge, a laccase-based Sunset Yellow biosensor has not previously been reported. Most of the previous studies focused on laccase-based biosensor for the detection of phenolic compounds [25,26].

## 2. Materials and Methods

### 2.1. Apparatus and Reagents

Laccase (E.C. 1.10.3.2) (0.5 µg, Sigma Aldrich, St. Louis, MO, USA), Sunset Yellow (98%, Sigma Aldrich), acrylamide (98%, Sigma Aldrich), ethyl methacrylate (97%, Sigma Aldrich), 2,2-dimethoxy-2-phenylacetophenone (99%, Sigma Aldrich), potassium dihydrogen phosphate (99%, Systerm, Shah Alam, Malaysia), alumina (Autolab, Ultrecht, The Netherlands), and dipotassium hydrogen phosphate (99%, Systerm) were used as received without further purification. The biosensor response was measured using a potentiostat (DropSens, Asturias, Spain) and GCE (Autolab).

### 2.2. Preparation of Poly(AAm) and Poly(AAm-co-EMA)

Preparation of poly(AAm) and poly(AAm-*co*-EMA) were conducted using a photopolymerization technique by ultraviolet exposure unit (UV). The poly(AAm-*co*-EMA) membranes used in the present study were composed of 90% (*w/w*) AAm and 10% (*w*/*w*) EMA. An amount of 100% (*w/w*) of AAm was used for poly(AAm) membrane and 1.6% *w/w* of 2,2 dimethoxy-2-phenylacetophenone (DMPP) photoinitiator was added into monomer mixture. For the preparation of the membrane, 10 mL of monomer mixture was loaded on the petri dish and exposed to ultraviolet radiation for 10 min in atmospheric gaseous nitrogen. As a result, a thin film of polymer was produced.

### 2.3. Preparation of Biosensor Membrane

Preparation of a copolymer membrane for the immobilization of laccase was conducted using a photopolymerization technique using an ultraviolet exposure unit (UV). A host monomer, AAm, was mixed with EMA to increase the hydrophilicity of AAm monomers. The poly(AAm-*co*-EMA) membranes used in the present study were composed of 90% AAm and 10% EMA. First, 0.9 g of AAm powder was dissolved in 799 µL of deionized water. Subsequently, 100 µL of EMA was added dropwise into the AAm solution. Then, 1.6% *w/w* of 2,2-dimethoxy-2-phenylacetophenone (DMPP) was added to the mixture.

Additionally, 10 mg/mL of laccase solution was prepared, which was then pipetted into 10 μL of the monomer mixture. The bare GC electrode was polished with alumina slurry until a mirror-like surface was evident. It was then washed with anhydrous alcohol and distilled water in an ultrasonic bath for 3 min and then dried via nitrogen gas blowing [17]. The active surface of a GCE (0.20 cm^2^ surface area) was dripped with 10 μL of the 1:1 ratio of laccase:monomer solution and then exposed to ultraviolet radiation (60 W) for 10 min under a nitrogen atmosphere [19].

### 2.4. Water Absorption Test

Water absorption test for poly(AAm) and poly(AAm-*co*-EMA) membranes were measured by immersing these membranes in different beakers. Each beaker contained distilled water at room temperature. In every two minutes, the membranes were weighed before and after the immersion and calculated by using an Equation (1) below [27]:

(1) $$\mathrm{Percentage}\;\mathrm{of}\;\mathrm{water}\;\mathrm{absorption}\;\left(\%\right)=\frac{W_{e}-W_{o}}{W_{o}}\;\times \;100\%$$

where W_e_ = weight of swollen polymer, g; W_o_ = weight of dry polymer, g.

### 2.5. Electrochemical Measurement of Sunset Yellow Using Laccase Immobilized in a Poly(AAm-co-EMA) Membrane

Cyclic voltammetry (CV) was carried out using a DropSens potentiostat to investigate the electrochemical properties of Sunset Yellow. The CV electrochemical cell potential was fixed from −1.500 to 1.500 V and 0.150 to 0.400 V, with a 0.05 V/s scan rate. A GC electrode coated with laccase immobilized into poly(AAm-*co*-EMA) was used as the working electrode. A GCE (Metrohm) was used as the auxiliary electrode, and Ag/AgCl (3 M KCl) was used as the reference electrode. The working electrode was placed between the other two electrodes in a beaker containing 0.05 M phosphate buffered saline (PBS) (pH 5).

### 2.6. Optimization of Poly(AAm-co-EMA)/Lac/GCE Biosensor Membrane

The optimization of parameters such as pH, enzyme loading, accumulation time and stability of the biosensor was conducted using differential pulse voltammetry (DPV). The electrochemical cell potential was fixed from 0.150 to 0.400 V with a scan rate of 0.05 V/s. A GCE (Metrohm) was used as the auxiliary electrode, Ag/AgCl (3 M KCl) as the reference electrode, and poly(AAm-*co*-EMA)/Lac/GCE as the working electrode. These three electrodes were immersed in a beaker containing 10 mL of buffer solution and 10 μM Sunset Yellow; the analyte concentration was fixed for all of the experiments. The effects of pH were studied by conducting measurements across a pH range of 1–9. Enzyme loadings of 0.025–0.500 mg/cm^2^, accumulation times of 0–5 min and stability of biosensor up to 21 days were studied.

### 2.7. Linear Range and Limit of Detection

Linear range was determined under optimal conditions with a Sunset Yellow concentration range of 0.08–30.00 μM. To determine the detection limit, a buffer solution was used as a blank. Both of the DPV experiments were conducted from 0.15 to 0.40 V with a scan rate of 0.05 V/s using a Dropsens potentiostat.

### 2.8. Interference Study of Biosensor

Interference study for Sunset Yellow biosensor was carried out by using several substances such as citric acid, glucose, ascorbic acid, and tartrazine. These substances are usually being added together with Sunset Yellow in soft drinks. The concentration of Sunset Yellow used was 6.00 μM and 1-, 10-, 50-, or 100-fold concentration of citric acid, glucose, ascorbic acid, or tartrazine was added to the solution. Biosensor response towards Sunset Yellow without the presence of interfering compounds also was recorded to obtain the percentage of interference when the interfering substances were present.

### 2.9. Determination of Sunset Yellow in Soft Drink

An electrode fabricated with poly(AAm-*co*-EMA)/Lac was used for the quantitative determination of Sunset Yellow in a soft drink purchased from a local market. First, 50 mL of the soft drink was boiled to remove carbon dioxide [20] before being diluted with buffer solution. Then, 10 µL of the treated sample was added to a buffer solution in a 10-mL beaker. Sunset Yellow was determined using the DPV method under optimal conditions. Recovery tests were performed with 0.08–2.00 μM Sunset Yellow. The concentration of Sunset Yellow was validated using an HPLC method [28].

## 3. Results and Discussion

### 3.1. Characterization of Poly(AAm) and Poly(AAm-co-EMA) by Fourier-Transform Infrared Red (FTIR)

**Figure 1:**
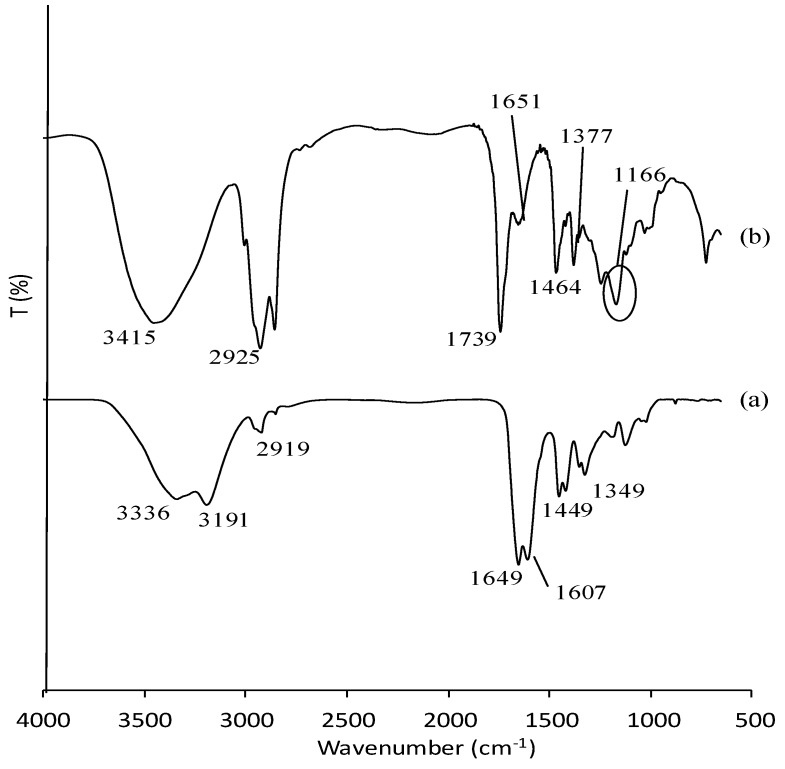
Fourier-Transform Infrared Red (FTIR) spectra of (**a**) poly(AAM) and (**b**) poly(AAM-*co*-EMA). Reproduced from [AIP Conference Proceedings 1784, 030017 (2016); doi:10.1063/1.4966755] with permission of AIP Publishing [32].

Figure 1 shows the FTIR spectra of poly(AAm) and poly(AAm-*co*-EMA). Poly(AAm) and poly(AAm-*co*-EMA) showed peaks at 3336, 3191 and 3415 cm^−1^ which attributed to the primary amine stretch [29,30]. The peak that corresponded to the stretching of –CH functional group at 2925 cm^−1^ in poly(AAm-*co*-EMA) seem more intense compared to poly(AAm) at 2919 cm^−1^. The peaks for C=O was also observed at 1739 cm^−1^ and 1649 cm^−1^ for poly(AAm). In addition, peak near 1651 and 1607 cm^−1^ in both spectra were assigned to the –NH bend. In addition, the peaks at 1464 cm^−1^ and 1449 cm^−1^ associated with the stretching vibration of –CN of poly(AAm-*co*-EMA) and poly(AAm) [29]. The most important peaks that attributed to –CH_3_ and –CO functional groups in the copolymer were contributed by EMA at 1377 and 1166 cm^−1^. Pavia et al. (2010) [31] reported that peak of –CH_3_ and –CO functional groups usually have bending vibration of approximately 1375 cm^−1^ and 1300 cm^−1^–1000 cm^−1^. These two important peaks prove the copolymerization occurred as they only appeared in poly(AAm-*co*-EMA) spectra [32].

### 3.2. Morphological Characterization

**Figure 2:**
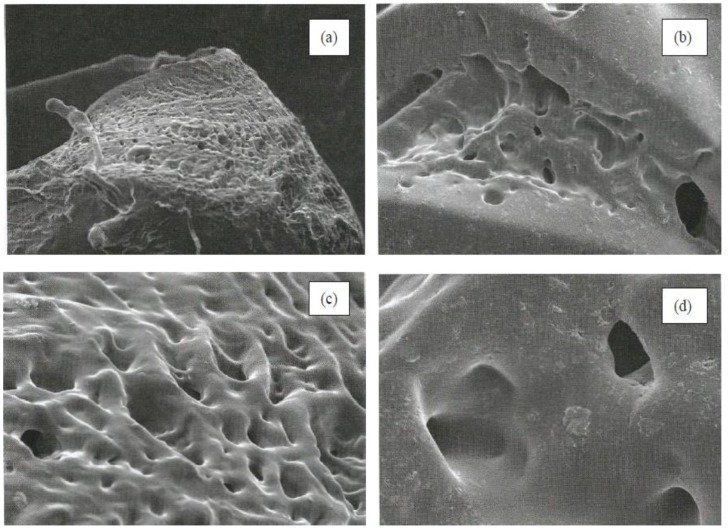
Micrographs of (**a**) poly(AAm) and (**b**) poly(AAm-*co*-EMA) at 100× of magnification, (**c**) Poly(AAm) and (**d**) poly(AAm-*co*-EMA) at 500× of magnification. Reproduced from [AIP Conference Proceedings 1784, 030017 (2016); doi:10.1063/1.4966755] with permission of AIP Publishing [32].

Figure 2 shows the micrographs of poly(AAm) and poly(AAm-*co*-EMA) at two different magnifications, 100× and 500×. There was good miscibility between AAm and EMA monomers. These micrographs also showed that poly(AAm) exhibited more porous surface. Poly(AAm-*co*-EMA) showed a smoother surface than poly(AAm). This finding was supported by Li et al. (2008) [33] and Gowda and Betageri (2011) [34] that poly(AAm) often has porous structure networks that will allow solute diffuse through the polymer structure [33].

### 3.3. Water Absorption Test

**Figure 3:**
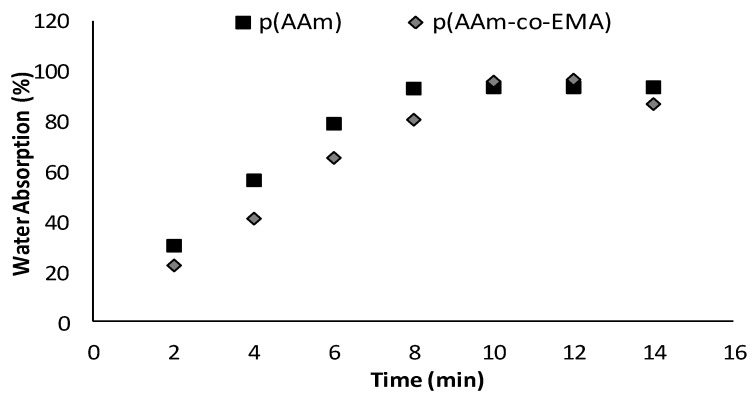
Water absorption percentage as a function of time for the membrane poly(AAm) and poly(AAm-*co*-EMA). Reproduced from [AIP Conference Proceedings 1784, 030017 (2016); doi:10.1063/1.4966755] with permission of AIP Publishing [32].

Figure 3 shows the water absorption percentage of poly(AAm) and poly(AAm-*co*-EMA) were approximately 94.0% and 96.2% respectively. Poly(AAm-*co*-EMA) had slightly higher water absorption percentage compared to poly(AAm). However, poly(AAm) required a shorter time (8 min) to reach equilibrium compared to poly(AAm-*co*-EMA) (10 min). Its means, an introduction of hydrophobic EMA monomer into poly(AAm) membrane was able to control the hydrophilicity properties of copolymer. Some degradation of both polymers also was observed in the distilled water and they had difficulty in maintaining original shape after reach the equilibrium state. Overall, water absorption for poly(AAm) membrane was slightly higher than poly(AAm-*co*-EMA) thus the highest ability to absorb water [32]. It had been reported that polar substitution groups such as –OH and –NH in a structure of polymer will cause problem in controlling the interaction of molecule with water-based solution. Amines group can ionize in media which are at pH below pK_b_ of ionization species and increasingly hydrophilic and highly swell [35]. Furthermore, the more porous surface of poly(AAm) also contributed to the higher water absorption. This will lead to enzyme leaching problem. It means poly(AAm-*co*-EMA) has better potential to be studied further for the application of enzyme immobilization and biosensor.

The poly(AAm-*co*-EMA) membrane was composed of 90.0% (*w/w*) AAm and 10% (*w/w*) EMA although water absorption test only 2.0% differences from poly(AAm). Further increase in EMA monomer cannot be tolerated in this study. It is because the deterioration of the sensitivities of the biosensors can be occurred with less hydrophilic membranes due to slow diffusion process. When the hydrophilicity is the lowest, the loss in sensitivity is thus the most severe [19]. Moreover, from the SEM image in Section 3.2, it clearly showed that poly(AAm-*co*-EMA) has smoother surface than poly(AAm). An amount of 10.0% of EMA monomer already covered most of the porous surface of the poly(AAm). Further increase in EMA monomer can cause more porous surface to be covered and contributed to lower water absorption as well as creating an interference to the analyte and contributed to a lower sensitivity of biosensor.

### 3.4. Electrochemical Behaviour of Sunset Yellow Using Poly(AAm-co-EMA)/Lac/GC Electrode

**Figure 4:**
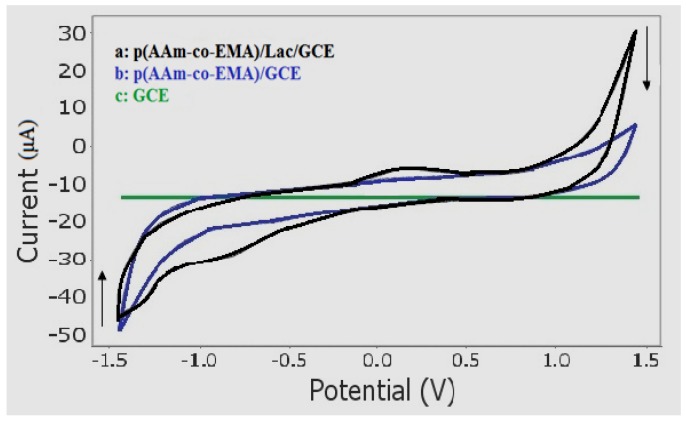
Cyclic voltammograms of Sunset Yellow 30 µM in 0.05 M and pH 5 of phosphate buffer solution at 5 mm diameter are (**a**) poly(AAm-*co*-EMA)/Lac/GCE working electrode, (**b**) poly(AAm-*co*-EMA)/GCE working electrode and (**c**) blank GCE working electrode with glassy carbon electrode as auxillary electrode and Ag/AgCl as reference electrode. S/N = 3 (S/N: ratio of mean to standard deviation of a measurement).

The electrochemical behavior of the Sunset Yellow biosensor was analyzed using 30 µM Sunset Yellow in 0.05 M PBS (pH 5) using a bare GC electrode (GCE), a poly(AAm-*co*-EMA) coated electrode, and an electrode modified with laccase (producing a poly(AAm-*co*-EMA)/Lac/GCE). The analyses were performed using CV with a potential window from −1.500 to 1.500 V. In the presence of Sunset Yellow, neither oxidation nor reduction peaks were observed from either the bare GCE or poly(AAm-*co*-EMA/GCE) (Figure 4). However, after laccase was introduced via a poly(AAm-*co*-EMA)/Lac membrane, a pair of well-defined and partially reversible redox peaks were observed in the cyclic voltammograms (I_pa_ = 2.132 µA, E_pa_ = 0.296 V; I_pc_ = −3.766 µM, E_pc_ = −0.806 V). These peaks indicated that the modified electrode significantly improved the redox reaction of Sunset Yellow, and therefore is suitable for use in a biosensor. As reported previously, anodic peak potentials of 0.6–0.95 V were recorded for Sunset Yellow [2,8,17]. However, the oxidation potential of the Sunset Yellow biosensor developed in the present study was lower than previously reported chemical sensors. Yang and Li [21] also reported a lower oxidation potential at 0.020 V (SCE references electrode). A biosensor with a low oxidation potential is favourable, because it can avoid oxidation of potential interferents that may coexist with Sunset Yellow in real samples. Sunset Yellow contains an –OH group attached to the benzene ring structure that accommodates an electron and facilitates proton transfer. This suggests that the redox reaction of Sunset Yellow occurred at the –OH group, with a potential of 0.296 V for oxidation and −0.806 V for reduction.

### 3.5. Effect of pH

**Figure 5:**
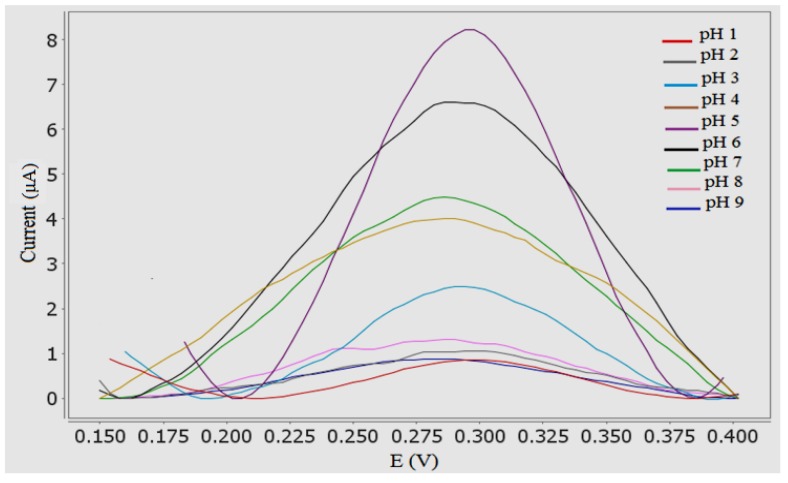
DPV for the effect of pH value on the oxidation peak potential of 10 µM SY in 0.05 PBS (pH range from 1 to 9). S/N = 3.

**Figure 6:**
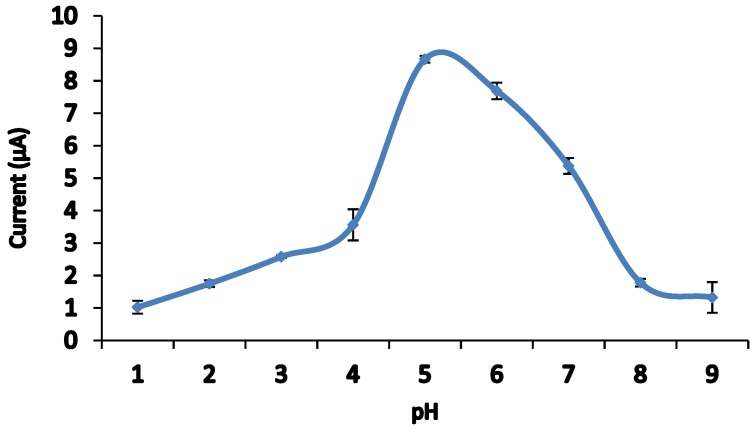
Effect of pH value on the oxidation peak potential of 10.00 µM SY in 0.05 PBS (pH range from 1 to 9). S/N = 3.

The electrochemical behavior of Sunset Yellow in 0.05 M PBS was studied across a range of pH values. The effect of pH on the response of the Sunset Yellow biosensor poly(AAm-*co*-EMA)/Lac/GCE was measured using DPV. Based on Figure 5 and Figure 6, the oxidation peak current increased from pH 1 to 4, and increased dramatically at pH 5. The current slowly decreased from pH 6 to 9. This decline was attributed to the loss of enzyme catalytic activity [23]. The Sunset Yellow signal was highest in pH 5 buffer; this condition was chosen for further studies. Similar results were reported by Li et al. [24], who entrapped laccase in silica spheres to detect dopamine in pH 5 PBS. In addition, these results correspond to the optimum pH range (3.5–5.0) of free laccase. Thus, the enzyme immobilization procedure was unlikely to affect the enzyme activity [36,37].

### 3.6. Influence of Laccase Loading

**Figure 7:**
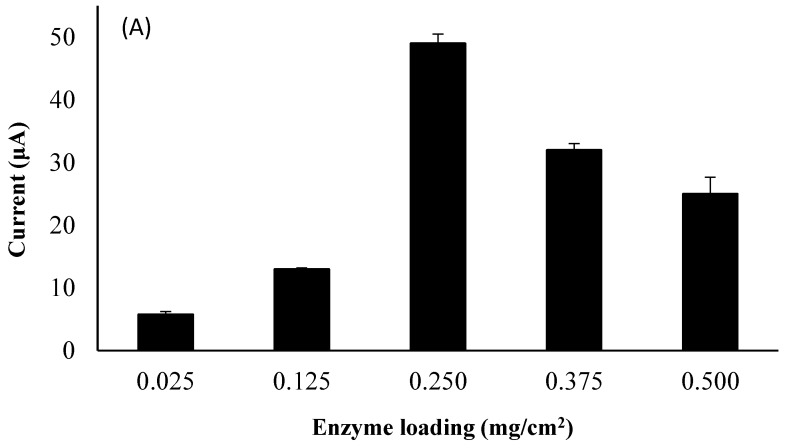

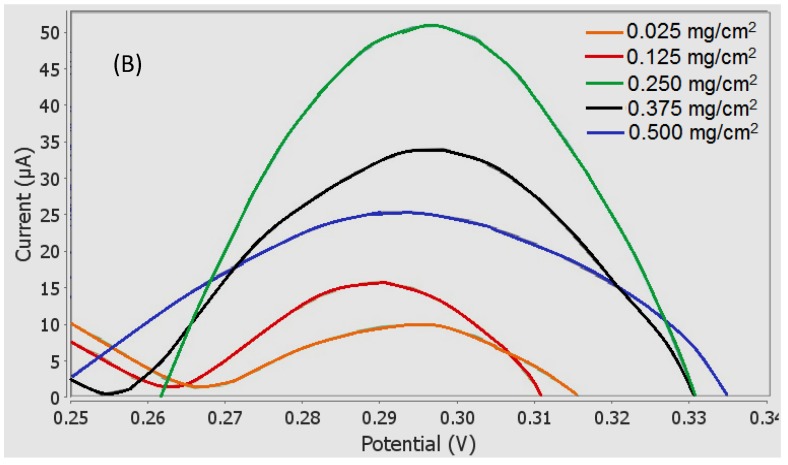
(**A**) Influence of Laccase enzyme loading in range 0.025 to 0.500 mg/cm^2^ on the oxidation peak currents of 10 µM SY in 0.05 M PBS. S/N = 3; (**B**) DPV for influence of laccase enzyme loading study.

To characterize the influence of laccase loading (0.025–0.500 mg/cm^2^) on the detection of Sunset Yellow, the electrochemical behavior of 10.00 µM Sunset Yellow in 0.05 M PBS was examined using poly(AAm-*co*-EMA)/Lac/GCE as a working electrode. It has been reported that the loading of immobilized enzyme on an electrode surface significantly affects biosensor sensitivity, detection limit, linear range, and substrate conversion [23]. As expected, the oxidation peak current of Sunset Yellow was strongly dependent on the enzyme loading (Figure 7A,B). Generally, the oxidation peak current increased with increasing enzyme loading. The maximum oxidation peak current was observed when the enzyme loading was 0.250 mg/cm^2^. However, increasing the enzyme loading from 0.250 to 0.500 mg/cm^2^ resulted in a significant decrease in the oxidation peak current of Sunset Yellow. This may be due to the photopolymerization technique, which results in a high-density crosslinked polymer with low interstitial space. Hence, the active site of the enzyme is also insulated, and not fully available to catalyze the redox reaction at high enzyme loadings, thereby decreasing laccase activity [28].

### 3.7. Effect of Accumulation Time

**Figure 8:**
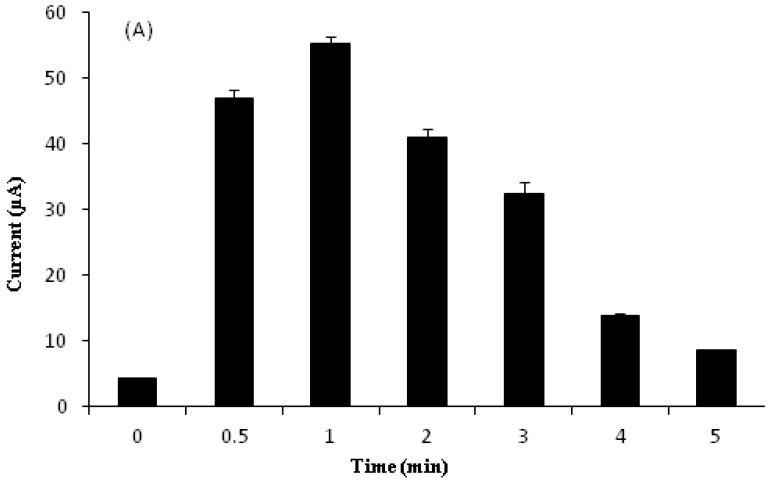

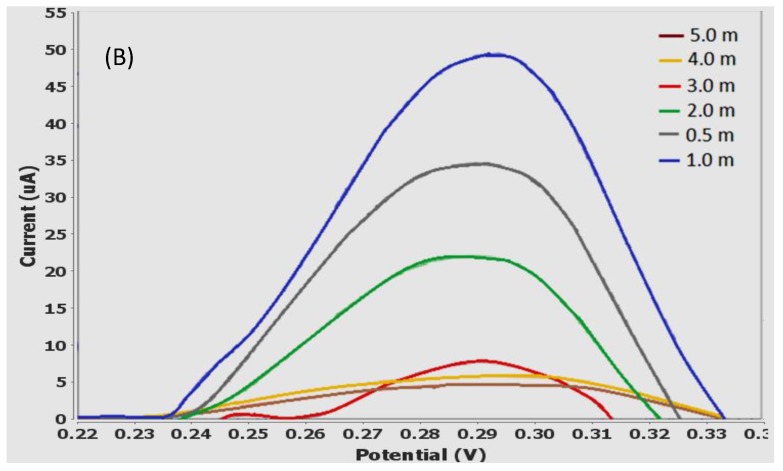
(**A**) Influence of accumulation time in range 0 to 5 min on the oxidation peak currents of 10.00 µM SY in 0.05 M PBS; (**B**) DPV for influence of accumulation time study.

Figure 8 shows the effects of accumulation time (0–5 min) on the oxidation peak current of 10 µM Sunset Yellow in 0.05 M PBS (pH 5) using a poly(AAm-*co*-EMA)/Lac/GCE. The signal increased dramatically from 0.5 to 1 min of accumulation time. Hence, accumulation time significantly affects biosensor sensitivity [21]. However, the oxidation peak current decreased when the accumulation time was increased from 1 to 5 min. This corresponds to previous findings, that the biosensor sensitivity may be limited by the saturation of Sunset Yellow on the electrode [5,7]. Therefore, an accumulation time of 1 min was chosen for use in further experiments.

### 3.8. Determination of Sunset Yellow

**Figure 9:**
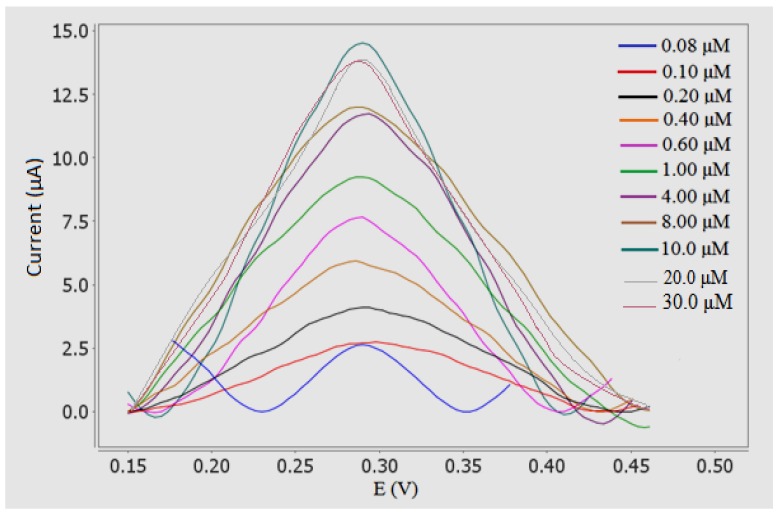
DPV of Sunset Yellow biosensor (polymer with different concentration of Sunset Yellow (in range from 0.08 to 30.0 µM) in 0.05 M PBS (pH 5) and accumulation time: 1 min (optimum condition) S/N = 3.

**Figure 10:**
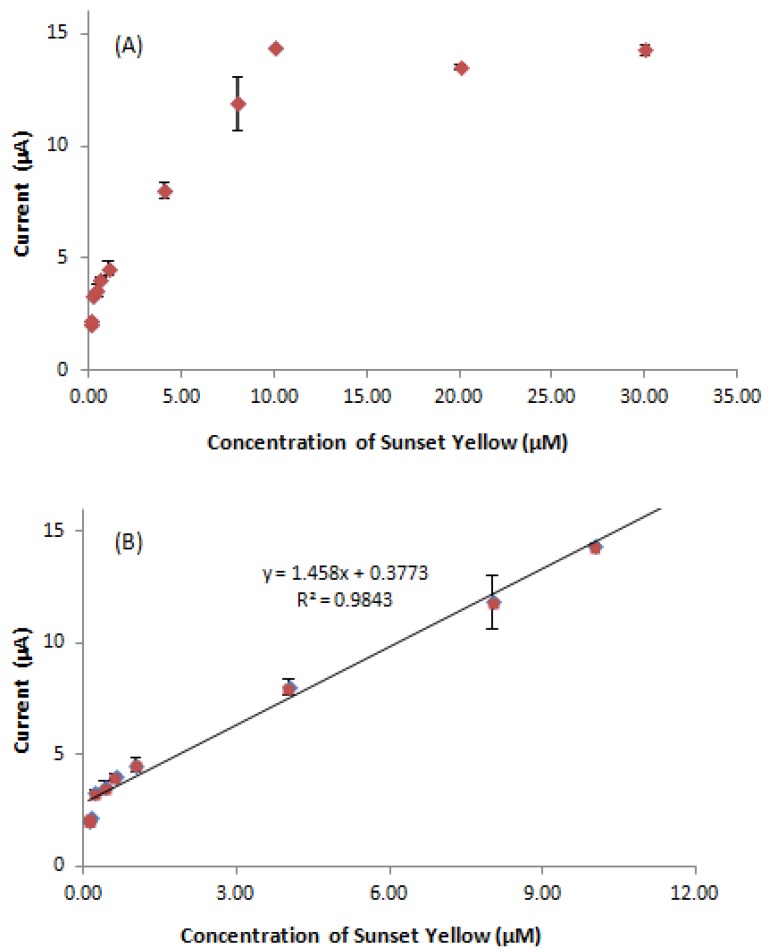
Calibration graph of Sunset Yellow biosensor (polymer with different concentration of Sunset Yellow (in range from (**A**) 0.08 to 30.00 µM and (**B**) 0.08 to 10.00 µM) in 0.05 M PBS (pH 5) and accumulation time: 1 min (optimum condition). S/N = 3.

Under the optimum condition, DPV was performed to investigate the relationship between the peak currents and different concentration of Sunset Yellow. The oxidation peak current increased with increasing Sunset Yellow concentration (Figure 9). In addition, the linear concentration range of Sunset Yellow was 0.08 to 10.00 µM (regression equation: *y* = 1.458*x* + 0.3773, *R*^2^ = 0.9843) (Figure 10B). At higher concentrations, the response was no longer linear because of the diffusion limit [38]. Yin et al. 2017 [39] also has stated that calibration curve gradually deviated from the straight line, indicating that saturated of adsorption was gradually reached. The detection limit (LOD) of Sunset Yellow was determined to be 0.02 μM. These results indicated a very good analytical performance of the developed electrochemical biosensor in food and beverages products analysis.

### 3.9. Comparison with Previous Reported Sunset Yellow Sensors

Some of the analytical characteristics obtained in the present study were compared to those from previous reports (Table 1). To date only this study that based on enzymatic biosensor to detect Sunset Yellow has been reported. Some novel electrochemical properties of Sunset Yellow were discovered in this work. Most of the reported findings on sensors commonly focused on designation of conductive materials based sensor to detect Sunset Yellow. They used conductive materials such as MWCNT, gold nanoparticles, graphene and conductive polymer in their sensor to promote direct electron transfer but has contributed to a higher oxidation potential for example 0.600–0.950 V [2,8,17,39,40]. At such potentials, sensor will detect interference from many other electroactive substances. In comparison, the oxidation of Sunset Yellow was dramatically shifted to lower potential using poly(AAm-*co*-EMA)/Lac/GCE biosensor. The decrease in overpotentials for Sunset Yellow oxidation was achieved due to extraordinary redox capability of laccase enzyme.

Moreover, compared to this study, most sensors in Table 1 used conductive material in order to improve sensitivity and lower detection limit of the sensors. The conductive materials have ability to assist better electron transfer process. From the fact, our biosensor has comparable result to these conductive material based chemical sensors (Table 1) although without the presence of conductive material, shows that biosensor has its own capability to oxidize Sunset Yellow. Biosensor also has exhibited better detection limit and sensitivity compared to gold nanoparticles and MWCNT based sensor reported by Ghoreishi et al. [2] and Rovina et al. [40]. In Table 1, although the sensors reported by Yu et al. [17] and Ye et al. [8] exhibited lower LOD than the sensor developed in the present study, their sensors had lower sensitivities. Furthermore, sensor reported by Chao and Ma [41] exhibited a higher LOD and sensitivity than the present biosensor. The LOD obtained in this study reached below the maximum level of Sunset Yellow in non-alcoholic beverages recommended by the European Commission 2011 (50 mg/L) [42]. This means the LOD value recorded in this study also still acceptable although much higher than LOD recorded in previous studies (Table 1). Overall, the LOD of our biosensor was comparable to previously reported findings, which indicates its feasibility for Sunset Yellow detection in real samples.

### 3.10. Reproducibility, Stability and Repeatability

The long-term stability of the modified electrode was evaluated through the DPV response of 10 µM Sunset Yellow. The poly(AAm-*co*-EMA)/Lac/GCE biosensor exhibited good reproducibility, repeatability, and stability. Five modified electrodes were used to measure 10 µM Sunset Yellow using the DPV method. The oxidation peak current of DPV was almost constant and the relative standard deviation (RSD) was 0.5–1.9%, confirming that the biosensor exhibited good repeatability. The RSD of Sunset Yellow (10 µM) determination using a poly(AAm-*co*-EMA)/Lac/GCE was 0.6–0.8% (*n* = 3). These results demonstrate that this biosensor has good repeatability and reproducibility. Moreover, the oxidation peak current for 10.00 µM Sunset Yellow was only reduced to 92.8% of its initial value after the modified GC electrodes were stored for seven days at 4 °C, indicating that the poly(AAm-*co*-EMA) matrix enhanced the stability and activity of immobilized laccase [44]. The similar electrode also being used to detect Sunset Yellow and biosensor response still retained up to 51.7% of its original value after 21 days of storage at 4 °C. It was due to the ability of the photocured hydrophilic membrane to retain water and hence maintaining the enzyme activity [45].

### 3.11. Interference Study

An interference study was conducted to ensure that other synthetic dyes, or substances such as citric acid, glucose, ascorbic acid, and tartrazine, would not interfere with Sunset Yellow detection in real samples. Therefore, 6 μM Sunset Yellow was prepared in 0.05 M PBS (pH 5), and a 1-, 10-, 50-, or 100-fold concentration of citric acid, glucose, ascorbic acid, or tartrazine was added to the solution [17]. Interferences were analyzed by DPV, and the results are shown in Table 2. Interference was calculated using the following formula: % interference = (*B*/*A*) × 100, where *B* is the current for the mixture containing Sunset Yellow and the interferent and *A* is the current for the Sunset Yellow solution. According to Thomas et al. [46] and Toffoli et al. [47], percentage interference of 90–110% is not considered to be significant. The average interferences for the tested species in Sunset Yellow solution were between 94.4% and 107.2%. Thus, the tested species did not interfere significantly in the detection of Sunset Yellow.

### 3.12. Sunset Yellow Determination in Soft Drink

**Figure 11:**
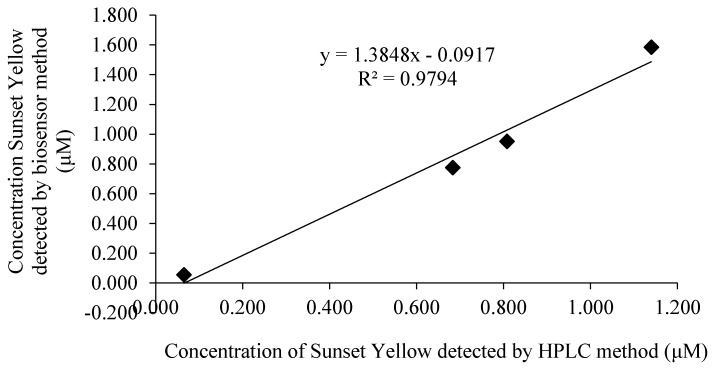
A comparison between the Sunset Yellow biosensor and HPLC method for the determination of Sunset Yellow.

As shown in Table 3, the analyzed soft drink contained 14.431 μM of Sunset Yellow, as determined by the immobilized laccase biosensor. Subsequently, the drink was spiked with 0.08, 0.8, 1, and 2 μM of Sunset Yellow to test biosensor recovery. The recovery percentage was 99–101.6%. The concentrations of Sunset Yellow were validated using HPLC, in order to determine the reliability of the biosensor (Table 4 and Figure 11). The result of Sunset Yellow determined by both methods were insignificant difference and a good correlation between the two methods was obtained (Figure 11). There was good agreement between the HPLC and biosensor results, suggesting that the biosensor is promising for in-situ detection applications.

## 4. Conclusions

A sensitive electrochemical method was developed for Sunset Yellow detections, based on a poly(AAm-*co*-EMA)/Lac/GCE system. The selection of poly(AAm-*co*-EMA) was carried out based on the morphology and swelling properties. The biosensor membrane coated onto the GCE effectively improved the redox response of Sunset Yellow, thereby increasing the sensitivity of the detection method. Under optimized conditions, the anodic peak current was linear in a Sunset Yellow concentration range of 0.08 to 10.00 µM; the detection limit was 0.02 μM. This biosensor showed excellent activity, which was retained to 92.8% after 7 days of dry storage at 4 °C. Furthermore, the poly(AAm-*co*-EMA)/Lac/GCE-based biosensor exhibited good reproducibility. The biosensor was validated in real samples using HPLC. The validation results were well correlated, and exhibited high sample recoveries. Hence, the laccase-based biosensor developed in this study is useful for the determination of Sunset Yellow in soft drinks, and is expected to facilitate the development of various electrochemical biosensors for detecting other food additives.

## Figures and Tables

**Table 1:** Comparison of electrochemical methods for Sunset Yellow detection.

Types of Matrix Used	pH	Potential (V)	Linear Range (µM)	LOD (µM)	Sensitivity (μA/μM)	References
Screen-printed electrode modified with gold nanoparticles.	4.0	0.750 (ox)	0.10–2.00	0.0300	1.490	[2]
β-cyclodextrin-layered poly(diallyldimethylammonium chloride)-graphene composite membrane.	5.0	0.820 (ox)	0.05–200.0	0.0120	0.476	[8]
Platinum nanoparticle and CTAB/graphene-composite-modified GCE.	3.0	0.811 (ox) *	0.08–10.00	0.0042	0.749	[17]
CTAB/Graphene/MWCNT-modified GCE.	6.0	−0.019 (ox) *	0.01–20.00	0.0100	0.260	[21]
Chitosan/CaONP/MWCNT/gold electrode.	7.0	1.00 (ox)	1.99–22.11	1.7685	1.326	[40]
Polydopamine-coated-MWCNT/GCE.	6.0	0.619 (ox) *	0.0022–4.64	0.0014	17.112	[39]
ZnONF/CPE.	5.0	0.691 (ox) *	0.001–0.02 0.02–0.15	0.0002	0.0046	[43]
Poly(AAm-*co*-EMA)/Lac/GCE.	5.0	0.296 (ox)	0.08–10.00	0.0200	1.458	This work

* ox: oxidation potential; * References [17,21,39,43] that used SCE as reference electrode, the potential values have been corrected by applied scale conversion between references electrodes.

**Table 2:** Interference in the presence of 6 μM Sunset Yellow.

Fold-Concentration of Interference Substance	Interference Percentage (%) ± RSD			
	Citric Acid	Glucose	Tatrazine	Ascorbic Acid
1	97.1 ± 1.23	95.2 ± 0.02	101.8 ± 0.140	106.6 ± 0.55
10	104.1± 0.38	98.5 ± 0.02	99.1 ± 1.26	100.4 ± 0.76
50	100.3 ±1.25	107.2 ± 0.19	105.9 ± 0.74	95.0 ± 0.44
100	97.8 ± 0.94	101.5 ± 0.25	96.3 ± 0.30	94.4 ± 1.82

**Table 3:** Determination and recovery of Sunset Yellow in soft drinks sample.

Sample	Spiked (μM)	Expected (μM)	Found (μM)	Recovery (%)
Soft Drink	0.00	-	14.431	-
	0.08	14.511	14.745	101.6
	0.80	15.231	15.466	101.5
	1.00	15.431	15.642	101.4
	2.00	16.431	16.274	99.0

**Table 4:** Validation of poly(AAm-*co*-EMA)/Lac/GCE biosensor with HPLC.

Sunset Yellow Concentration (μM)	HPLC Method (μM) ± SD	Biosensor Method (μM) ± SD	Relative Error (%)	Correlation Curve
0.08	0.065 ± 0.12	0.055 ± 0.60	18.18	*y* = 1.3848*x* ‒ 0.0917 *R*^2^ = 0.9794
0.80	0.683 ± 0.13	0.776 ± 0.60	−11.98	
1.00	0.808 ± 0.34	0.952 ± 0.64	−15.13	
2.00	1.140 ± 0.04	1.584 ± 1.20	−28.03	
